# The Association between the Risk of Aortic Aneurysm/Aortic Dissection and the Use of Fluroquinolones: A Systematic Review and Meta-Analysis

**DOI:** 10.3390/antibiotics10060697

**Published:** 2021-06-10

**Authors:** Chih-Cheng Lai, Ya-Hui Wang, Kuang-Hung Chen, Chao-Hsien Chen, Cheng-Yi Wang

**Affiliations:** 1 Department of Internal Medicine, Kaohsiung Veterans General Hospital, Tainan Branch, Tainan 710, Taiwan; n261@mail.vhyk.gov.tw; 2 Medical Research Center, Cardinal Tien Hospital and School of Medicine, College of Medicine, Fu Jen Catholic University, New Taipei City 242062, Taiwan; yhwang531@mospital.com; 3 Department of Internal Medicine, National Taiwan University Hospital, Taipei 100, Taiwan; khchen@ntuh.gov.tw; 4 Division of Pulmonary, Department of Internal Medicine, MacKay Memorial Hospital, Taipei 104217, Taiwan; 5 Department of Medicine, MacKay Medical College, New Taipei City 25245, Taiwan; 6 Department of Internal Medicine, Cardinal Tien Hospital and School of Medicine, College of Medicine, Fu Jen Catholic University, New Taipei City 23148, Taiwan

**Keywords:** fluroquinolone, aortic aneurysm, aortic dissection, azithromycin, sulfamethoxazole–trimethoprim, amoxicillin

## Abstract

This study aimed to investigate the association between the risk of aortic aneurysm (AA)/aortic dissection (AD) and the use of fluoroquinolones (FQs). PubMed, Embase, Cochrane CENTRAL, Cochrane Database of Systematic Reviews, Web of Science and Scopus were searched for relevant articles to 21st February 2021. Studies that compared the risk of AA/AD in patients who did and did not receive FQs or other comparators were included. The pooled results of nine studies with 11 study cohorts showed that the use of FQs increased the risk of AA/AD by 69% (pooled risk ratio (RR) = 1.69 (95% CI = 1.08, 2.64)). This significant association remained unchanged using leave-one-out sensitivity test analysis. Similar results were found for AA (pooled RR = 1.58 (1.21, 2.07)) but no significant association was observed for AD (pooled RR = 1.23 (0.93, 1.62)). Stratified by the comparators, the use of FQs was associated with a significantly higher risk of AA/AD compared to azithromycin (pooled RR = 2.31 (1.54, 3.47)) and amoxicillin (pooled RR = 1.57 (1.39, 1.78)). In contrast, FQ was not associated with a higher risk of AA/AD, when compared with amoxicillin/clavulanic acid or ampicillin/sulbactam (pooled RR = 1.18 (0.81, 1.73)), sulfamethoxazole–trimethoprim (pooled RR = 0.89 (0.65, 1.22)) and other antibiotics (pooled RR = 1.14 (0.90, 1.46)). In conclusion, FQs were associated with an increased risk of AA or AD, although the level of evidence was not robust. However, FQs did not exhibit a higher risk of AA or AD compared with other broad-spectrum antibiotics. Further studies are warranted to clarify the role of FQs in the development of AA or AD.

## 1. Introduction

Fluoroquinolone (FQ) is a broad-spectrum antibiotic with favorable oral bioavailability. Since the introduction of FQs, their use has rapidly increased [1], and they are one of the top three most prescribed classes of antibiotics at many sites [2,3,4]. However, safety issues are a concern when prescribing any medication. Regarding FQs, collagen-related adverse events such as tendon rupture and retinal detachment have been reported [5,6].

In addition, an animal study demonstrated that ciprofloxacin could increase the incidence of aneurysm formation, which was attributed to an increase in active matrix metalloproteinase 9 and decreased lysyl oxidase signaling [7]. Moreover, FQs have been reported to affect the level of circulating cytokines, such as interleukin 6, which was elevated in patients with abdominal aortic aneurysms [8]. Furthermore, several observational studies [9,10,11,12,13] have reported that the use of FQs was associated with an increased risk of aortic diseases including aortic aneurysm (AA) and aortic dissection (AD). In a population-based study of Taiwanese adults, Lee et al. showed that current use of FQ was associated with more than a twofold increased risk of AA or AD [10]. In another nationwide cohort study in Sweden, Pasternak et al. demonstrated that the rate of AA/AD among FQ users was 1.2 cases per 1000 person years, which was significantly higher than amoxicillin users (0.7 cases per 1000 person years) [12]. Moreover, meta-analyses of these studies [9,10,11,12] have confirmed the positive association between FQs and the risk of subsequent AA or AD [14,15,16,17]. These findings prompted the US Food and Drug Administration and the European Medicines Agency to issue safety warnings about the FQ-associated risk of AA or AD [18,19]. However, similar findings were not demonstrated in two recent studies [20,21], in which the authors adjusted for the potential confounding factors of coexisting infections and the indication for FQs. Therefore, we conducted this systematic review and meta-analysis to provide updated evidence and clarify the inconsistent findings with regards to this important issue.

## 2. Materials and Methods

### 2.1. Search Strategy

A literature search was performed following the PRISMA (Preferred Reporting Items for Systematic Reviews and Meta-Analyses) guidelines [22]. The protocol was registered at PROSPERO with the reference number CRD42020220007. Electronic databases including PubMed, Embase, Cochrane CENTRAL collecting secondary source, Cochrane Database of Systematic Reviews, Scopus and Web of Science—a meta-search engine—were searched for relevant studies published since inception (Pubmed from 1966; Embase from 1947; Web of Science from 1900; Cochrane from 1993; Scopus from 1970) to 21st February, 2020. The key search words were: fluoroquinolone (including besifloxacin, ciprofloxacin, delafloxacin, enrofloxacin, enoxacin, fleroxacin, gatifloxacin, gemifloxacin, levofloxacin, lomefloxacin, moxifloxacin, nemonoxacin, norfloxacin, ofloxacin, pefloxacin, sitafloxacin, sparfloxacin), quinolone, aortic dissection, aortic aneurysm and aortic dilatation. Details of the search strategy are described in Table S1. The reference lists of the relevant articles and Google Scholar were also searched manually to identify further studies. The literature search was limited to the English language.

### 2.2. Study Selection and Data Extraction

Two investigators (CCL and CHC) independently screened and reviewed each study. Studies were included if they met the following criteria: (1) the patients were aged ≥ 18 years; (2) FQs were used as the intervention; (3) a comparison group that did not receive an FQ or took another or no antibiotics were included; and (4) outcome of AD or AA. Conference abstracts and meta-analyses were excluded. A third reviewer (CYW) was consulted to resolve any disagreements.

### 2.3. Quality Assessment

Risk of bias was assessed using the ROBINS-I (Risk of Bias in Non-randomized Studies of Interventions) tool, which evaluates the quality of non-randomized studies [23]. Studies were rated as being “low risk”, “high risk” or “unclear” by two reviewers subjectively according to seven domains, including bias due to confounding, bias in selection of participants into the study, bias in classification of interventions, bias due to deviations from the intended interventions, bias due to missing data, bias in measurement of outcomes and bias in selection of the reported result. We also used the grading of recommendations assessment, development and evaluation (GRADE) methodology to rate the quality of the evidence for primary outcome as high, moderate, low or very low [24]. Observational studies using ROBINS-I tool began as high-quality evidence, which could be rated down because of risk of bias, inconsistency, indirectness, imprecision and publication bias and rated up because of large effect, dose response and all plausible residual confounding [25,26,27,28,29,30,31]. Two reviewers subjectively reviewed all included studies for risk of bias and all outcomes for quality of evidence. The third reviewer was consulted if there was any disagreement between the two reviewers.

### 2.4. Outcome Measure and Statistical Analysis

The primary outcome of interest was AA and/or AD. Rate ratios, hazard ratios and odds ratios from individual studies were extracted for meta-analysis. The average effects were calculated to combine data across study arms. If there were multiple study cohorts in one study, the data was presented and analyzed separately. Risk ratios (RR) were considered as a measure of effect size of meta-analysis for the association between the use of FQs and AA/AD. Statistical significance was considered if the 95% confidence interval (95% CI) did not include 1 for the RR.

Pooled RRs across studies were calculated using a Der–Simonian–Laird random effects model [32]. Initially, we assumed that the effect size is not identical across studies due to different patient profiles, comparator drugs, study designs and outcome definition; that is, the effect size comes from a distribution of true effect sizes. As a result, the between-study variance (i.e., tau-squared) was considered in the random-effects model. Secondly, the study weights were more similar in the random-effects model so that the effects of studies with small sample size would not be ignored [33]. A two-sided *p* value of <0.05 was considered to be significant. Study heterogeneity was presented using χ^2^-based Cochran’s Q and I2 statistics [34,35]. For the Q statistic, *p* values < 0.10 was considered as statistically significant for heterogeneity. For the I2 statistic, heterogeneity was assessed as follows: low heterogeneity (I2 = 0–40%), moderate heterogeneity (I2 = 30–50%), substantial heterogeneity (I2 = 50–90%) and considerable heterogeneity (I2 = 90–100%) [36]. Leave-one-out sensitivity analysis was performed by excluding one study at a time to evaluate whether a single study had a large influence on the main pooled results. Subgroup analyses were performed to evaluate whether the results differed according to comparators, study design, age and sex. To evaluate publication bias, funnel plots for the primary outcome with effect sizes plotted against their standard errors were presented. Egger’s regression intercept method was used to examine the asymmetry of the funnel plots; the regression intercept was zero in the absence of publication bias [37]. All statistical analyses were performed using Comprehensive Meta-Analysis Version 3 (Biostat, Englewood, NJ, USA).

## 3. Results

### 3.1. Literature Search and Evaluation for Study Inclusion

**Figure 1:**
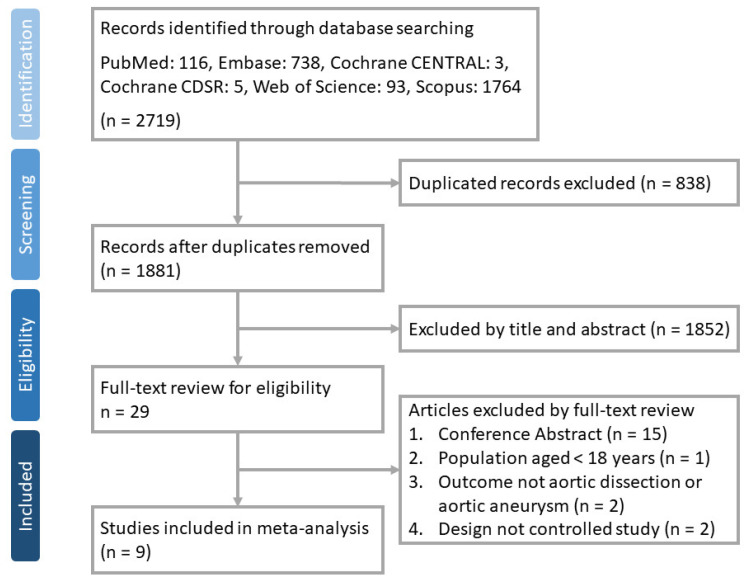
Flow chart of study selection.

A total of 2719 articles were identified from PubMed (*n* = 116), Embase (*n* = 738), Cochrane CENTRAL (*n* = 3), Cochrane Database of Systematic Reviews (*n* = 5), Web of Science (*n* = 93) and Scopus (*n* = 1764). Twenty-nine articles were selected after removing duplicate records (*n* = 838) and ineligible ones by title and abstract review (*n* = 1852). A total of nine studies [9,10,11,12,13,20,21,38,39] were included after removing 20 articles after full-text review (Figure 1).

### 3.2. Study Characteristics

Table 1 summarizes the characteristics of the nine included studies [9,10,11,12,13,20,21,38,39]. Three studies each were conducted in Taiwan and the US, and one each in Canada, France and Sweden. The study designs, inclusion criteria, follow-up periods and comparators varied.

### 3.3. Quality Assessment

**Figure 2:**
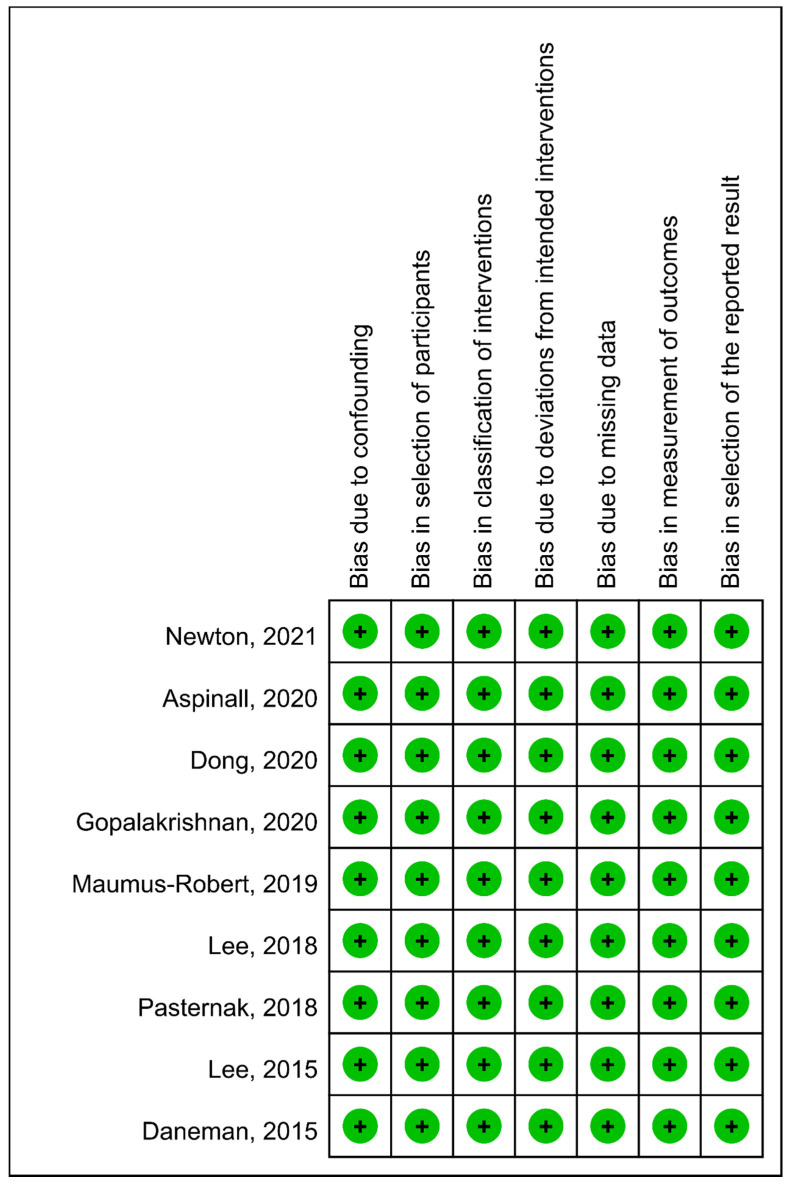
Summary of risk of bias.

The assessment of risk of bias is presented in Figure 2. All the nine studies had a low risk of bias according to study design, data collection and analyses. The quality of the evidence for the outcome of AA/AD using GRADE methodology was rated as moderate due to inconsistency.

### 3.4. Outcome Analysis

**Figure 3:**
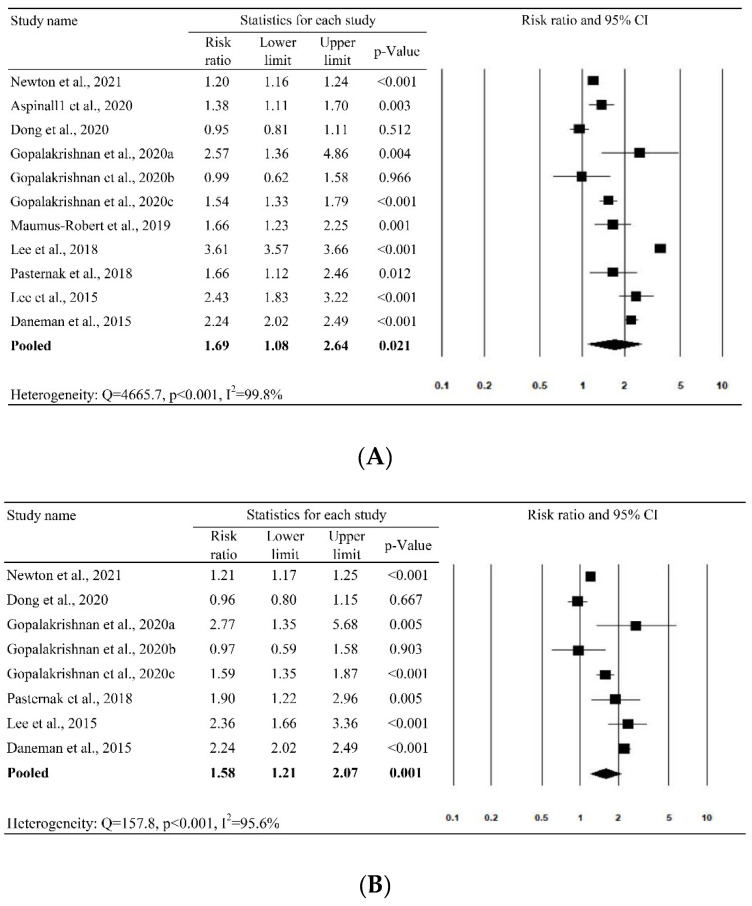

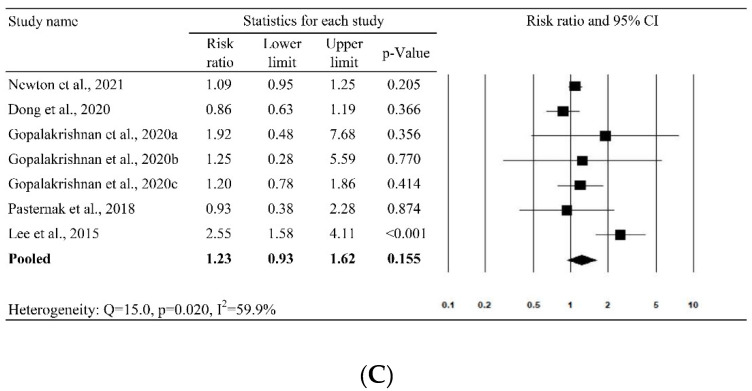
Forest plot of the risk of (**A**) aortic aneurysm/aortic dissection, (**B**) aortic aneurysm and (**C**) aortic dissection.

The nine studies with 11 study cohorts were included in the meta-analysis for the outcome of AA/AD. The pooled results show that the use of FQ increased the risk of AA/AD by 69% (pooled RR = 1.69 (95% CI = 1.08, 2.64, 95% prediction interval [PI] = 0.29, 9.70)), even though the heterogeneity across studies was high (Q = 4665.7, *p* < 0.001, I2 = 99.8%) (Figure 3A). Similar results were found for AA (pooled RR = 1.58 (95% CI = 1.21, 2.07; 95% PI = 0.63–3.97), Figure 3B) but no significant association was observed for AD (pooled RR = 1.23 (95% CI = 0.93, 1.62; 95% PI = 0.58, 2.61), Figure 3C).

### 3.5. Sensitivity Analysis

**Figure 4:**
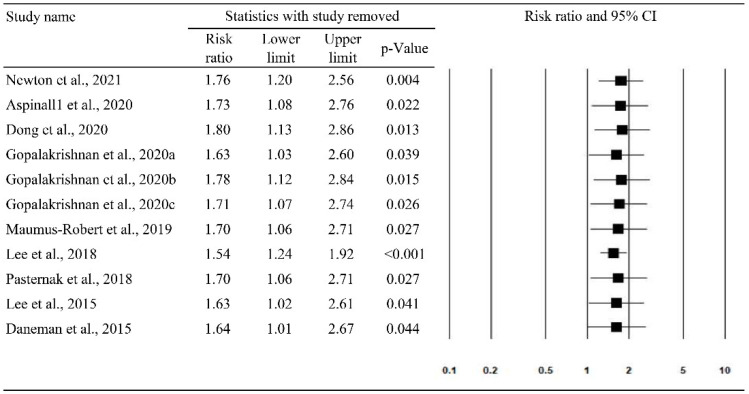
The leave-one-out sensitivity analysis for aortic aneurysm/aortic dissection.

Leave-one-out sensitivity analysis is shown in Figure 4. The results show that no study had a large influence on the main results for the association between the use of FQs and AA/AD, since the magnitude and direction of the associations did not change when including studies that had been removed one at a time.

### 3.6. Publication Bias

**Figure 5:**
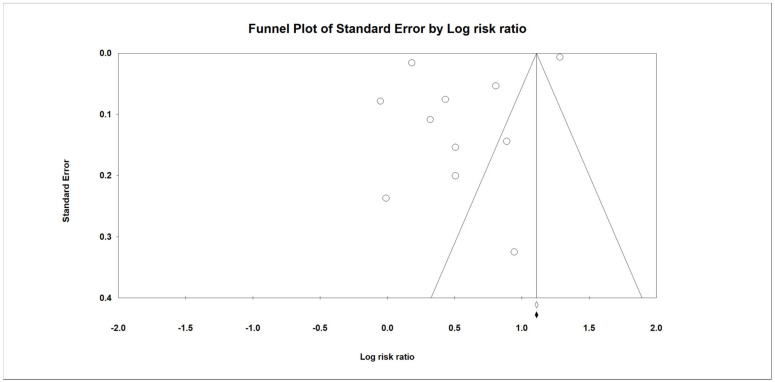
Publication bias in funnel plot for aortic aneurysm/aortic dissection.

Figure 5 shows the funnel plot representing the effect size of AA/AD against the standard error. Egger’s test (intercept = −10.3, t = 1.41, df = 9, *p* = 0.192) was not significant, suggesting no publication bias. However, the funnel plot was asymmetric so the possibility of publication bias still could not be ruled out.

### 3.7. Subgroup Analysis

Table 2 summarizes the results of the subgroup analysis. First, to examine whether the study design of the included studies was a factor related to the high heterogeneity, we performed subgroup analysis according to the study design. The results show that FQ was associated with a significantly higher risk of AA/AD than their counterparts in case-time-control studies (pooled RR = 2.49 (1.16, 5.32)) and cohort studies (pooled RR = 1.59 (1.16, 2.18)). In contrast, no significant association was observed in the subgroup analysis of nested case control studies (pooled RR = 1.51 (0.60, 3.75)). Second, the significant association between FQ use and the risk of AA/AD was observed for both sexes—female (pooled RR = 1.79 (1.13, 2.83)) and male (pooled RR = 1.32 (1.12, 1.55)). Third, subgroup analysis according to age group found that FQ was associated with the risk of AA/AD among patients aged 50–64 years (pooled RR = 1.24 (1.20, 1.29)), but not for patient aged ≥ 65 years (pooled RR = 1.51 (0.77, 2.96)). Fourth, to avoid potential surveillance bias, we performed a subgroup analysis of the patients with baseline image and did not find a significant association between FQ use and the risk of AA/AD (pooled RR = 1.05, (0.91–1.21)) in this subgroup.

According to the type of infection, no significant association between FQ use and the risk of AA/AD was observed in the subgroups with lower respiratory tract infection/pneumonia (pooled RR = 1.58 (0.68–3.69)) and urinary tract infection (pooled RR = 0.80 (0.58–1.10)). Stratified by the comparators, the use of FQs was associated with a significantly higher risk of AA/AD compared to azithromycin (pooled RR = 2.31 (1.54, 3.47)) and amoxicillin (pooled RR = 1.57 (1.39, 1.78)). In contrast, FQ was not associated with a higher risk of AA/AD, when compared with amoxicillin/clavulanic acid or ampicillin/sulbactam (pooled RR = 1.18 (0.81, 1.73)), sulfamethoxazole–trimethoprim (pooled RR = 0.89 (0.65, 1.22)) and other antibiotics (pooled RR = 1.14 (0.90, 1.46)).

## 4. Discussion

In this meta-analysis, we reviewed nine studies to assess the association between the risk of AA/AD and the use of FQs. First, the pooled analysis of the nine studies showed that exposure to FQs was associated with an increased risk of AA or AD (RR, 1.69; 95% CI: 1.08–2.64; I2 = 99.8%). Moreover, this association between FQs and AA/AD remained consistent in the leave-one-out sensitivity analysis. In addition, increased risks of AA (RR, 1.58; 95% CI: 1.21–2.07; I2 = 95.6%) following the use of FQs were demonstrated in the subgroup analysis. These findings are consistent with previous meta-analyses [14,15,16,17,40]. Even in the subgroup analysis, this significant association between FQs and AA/AD remained in the patients aged 50–64 years and in both genders. Moreover, with regards to the study designs, this association remained significant in the analysis of case-time-control and cohort studies, but it became insignificant in the analysis of nested case-control studies. Although most of the findings in this meta-analysis suggest a possible association between the use of FQs and the development of AA/AD, there is still concern about the results because the included studies had high heterogeneity (most I2 > 50%) and the findings of the asymmetric funnel plot indicated possible publication bias.

Even though the findings indicated that exposure to FQs may be associated with a higher risk of AA/AD, we were concerned as to whether this association was caused by underlying infections or FQs themselves. All data included in this meta-analysis were from observation studies, and the selection of appropriate antibiotics should be according to the site of infection and disease severity. Clinically, amoxicillin and azithromycin would be only prescribed for patients with mild infections, and FQs and other broad-spectrum antibiotics would be prescribed for patients with moderate or severe infections. First, FQ was not associated with a higher risk of AA/AD than the comparators in the subgroup analysis of patients with lower respiratory tract infection/pneumonia and urinary tract infection. Second, we performed further subgroup analysis according to the use of antibiotics and found that the risk of AA/AD was higher in the FQ users compared to those who received amoxicillin and azithromycin. In contrast, the use of FQs was associated with a similar risk of AA/AD compared to other antibiotics, including amoxicillin/clavulanic acid or ampicillin/sulbactam, and other broad-spectrum antibiotics. Therefore, our findings may imply that the development of AA/AD may be related to the severity of the infection, but not FQs themselves.

Our study has several strengths. We included nine studies, which is more than previous meta-analyses [14,15,16] which included fewer than five studies on the same subjects. Therefore, we could obtain more data to analyze and provide more solid evidence than previous meta-analyses [14,15,16]. Moreover, we also performed sensitivity analysis and various subgroup analyses to validate our findings.

There are also some limitations to our meta-analysis. First, no randomized controlled studies on this issue were found, and all the selected studies were observational. However, most of the selected studies adjusted for confounders by using either propensity score matching [12,21], propensity score adjustment [10], risk-set sampling [20], adjustment for time-varying confounders [9], adjustment for baseline characteristics and indications of FQ [11,13,21]. Second, the possible roles of infectious pathogens in the development of aortic structural abnormalities including aortic aneurysm and aortic aneurysm rupture were not investigated in this study. Third, the asymmetry of our funnel plots suggested the existence of publication bias. Based on the finding that the pooled RR moved toward the null after including two imputed studies with negative results and small standard errors, we cannot exclude the possibility that the observed association may have been over-estimated. Finally, the heterogeneity remained very high, although we performed subgroup analyses according to various factors related to aneurysm/dissection and the patients’ characteristics. One of the reasons may be that the patients included in individual studies were very heterogenous. Further research is warranted to identify potential factors that may have affected consistency across the studies, such as severity of aneurysm/dissection, indications for the use of FQs or antibiotics, dosage of FQs and duration of response.

In conclusion, the use of FQs was associated with an increased risk of AA/AD. However, compared with other broad-spectrum antibiotics, FQs had a similar risk of AA/AD, suggesting that the risk of AA/AD could be related to the underlying severity of disease but not antibiotics themselves. However, further prospective studies are warranted to clarify the role of FQs in the development of AA/AD after adjustment for underlying infection and its severity.

## Figures and Tables

**Table 1:** The characteristics of included studies.

Reference	Study Design	Study Location	Follow-Up Duration	Current FQ Exposure	Control Group	Case No.		Age, Year *		Male, %		
						Study Group	Control Group	Study Group	Control Group	Study Group	Control Group	Primary Outcome
Daneman et al. 2015	Population-based longitudinal cohort study	Ontario, Canada	Range: 2–17 years	Within 30 days before event	FQ non-users	657,950 FQ user	1,086,410	65	65	48.6	48.9	Severe collagen- associated adverse event including AA
Lee et al. 2015	Nested case-control study	Taiwan	Mean: 3613.3 days	Within 60 days before event	Not hospitalized for AA or AD	1477 AA or AD	147,700	74.7 ± 11.7 (AA); 66.2 ± 14.5 (AD)	71.0 ± 13.7	74.1 (AA); 71.5 (AD)	72.9	AA or AD
Pasternak et al. 2018	Nationwide cohort study with active comparator	Sweden	120 days	Within 60 days before event	Amoxicillin	360,088 episodes of FQ use	360,088	67.9 ± 10.8	68.0 ± 10.4	45	45	AA or AD
Lee et al. 2018	Case crossover study	Taiwan	300 or 60–180 days	Within 60 days before event	As their own control during the reference period	1213 hospitalized AA/AD		70.6 ± 13.8	70.6 ± 13.8	72.5	72.5	AA or AD
Maumus-Robert et al. 2019	Case-time-control study	France	180 days	Within 30 days before event	As their own control window (day 120–180 before event)	5946 AA or AD		NA	NA	NA	NA	AA or AD
Dong et al. 2020	Nested case-control study	Taiwan	1303.82 days	Within 60 days before event	Free of AA/AD at the time a case occurred	28,948	289,480	67.4 ± 15.0	67.4 ± 15.0	71.4	71.4	AA or AD
Gopalakrishnan et al. 2020	PMS case-control cohort study	US	NA	Within 60 days before event	AZM, SMX/TMP, AMX	139,772 (PN); 474,182 (UTI)	139,772 (PN); 474,182 (UTI)	63.7 ± 11.0 (PN); 62.1 ± 10.4 (UTI)	63.6 ± 11.0 (PN); 62.0 ± 10.3 (UTI)	46.4 (PN); 13.3 (UTI)	46.3 (PN); 13.0 (UTI)	AA/AD
Aspinall et al. 2020	Self-controlled case series	US	NA	Within 30 days before event	AMX, AZM, CXM, CFX, DOX, SMX/TMP	2027 (total); 88,606 (person days)	2027 (total); 120,804 (person days)	68.8 ± 8.8	68.8 ± 8.8	98.3	98.3	AA/AD
Newton et al. 2021	Population-based cohort study	US	NA	Within 180 days before event	AMC, AZM, CFX, CLI, SMX/TMP	9,053,961	38,542,584	44 (32–55)	44 (32–55)	39.1	40.1	AA/AD

AA, aortic aneurysm; AD, aortic dissection; NA, not applicable; PN, pneumonia; UTI, urinary tract infection; SMX/TMP, sulfamethoxazole/trimethoprim; AMX, amoxicillin; AZM, azithromycin; CXM, cefuroxime; CFX, cephalexin; DOX, doxycycline; AMC, amoxicillin–clavulanate; CLI, clindamycin; FQ, fluoroquinolone. * presented as mean ± SD or median (IQR).

**Table 2:** Subgroup analyses.

Characteristics	Study No.	Risk Ratio	95% CI	*p*-Value
Study design				
Case-time-control study	2	2.49	1.16–5.32	0.019
Cohort study	6 cohorts in 3 studies	1.59	1.16–2.18	0.004
Nested case-control study	2	1.51	0.60–3.78	0.382
Sex				
Female	4	1.79	1.13–2.83	0.013
Male	5	1.32	1.12–1.55	0.001
Age group				
50–64 years	2	1.24	1.20–1.29	<0.001
≥65 years	3	1.51	0.77–2.96	0.227
Patients with baseline image	2	1.05	0.91–1.21	0.521
Type of infection				
Lower respiratory tract infection/pneumonia	2	1.58	0.68–3.69	0.284
Urinary tract infection	2	0.80	0.58–1.10	0.168
Comparator				
Azithromycin	2	2.31	1.54–3.47	<0.001
Amoxicillin	3	1.57	1.39–1.78	<0.001
Amoxicillin/clavulanate or ampicillin/sulbactam	2	1.18	0.81–1.73	0.384
Sulfamethoxazole/trimethoprim	2	0.89	0.65–1.22	0.462
Other antibiotic	3	1.14	0.90–1.46	0.284

CI, confidence interval.

## Data Availability

The data presented in this study are openly available on the studies referenced in the figures, and individual data of each can be consulted in the original manuscripts.

## Supplementary Materials

The following are available online at https://www.mdpi.com/article/10.3390/antibiotics10060697/s1, Table S1: Search strategy.
