# Supplementary material for: The Association between the Risk of Aortic Aneurysm/Aortic Dissection and the Use of Fluroquinolones: A Systematic Review and Meta-Analysis
Source: Antibiotics (Basel). 2021 Jun 10;10(6):697. doi: 10.3390/antibiotics10060697 (PMC8230555; doi:10.3390/antibiotics10060697)
Supplement: Supplementary file 1 [file antibiotics-10-00697-s001.zip › antibiotics-1234025-supplementary.pdf]

antibiotics

MDPI

Online Supplemental material to:

Review

The Association between the Risk of Aortic Aneurysm/Aortic Dissection and the Use of Fluroquinolones: A Systematic Review and Meta-Analysis

Chih-Cheng Lai [1], Ya-Hui Wang [2], Kuang-Hung Chen [3], Chao-Hsien Chen [4,5,*] and Cheng-Yi Wang [6,*]

[1] Department of Internal Medicine, Kaohsiung Veterans General Hospital, Tainan Branch, 710, Tainan, Taiwan; n261@mail.vhyk.gov.tw
[2] Medical Research Center, Cardinal Tien Hospital and School of Medicine, College of Medicine, Fu Jen Catholic University, 231 New Taipei City, Taiwan; yhwang531@mospital.com
[3] Department of Internal Medicine, National Taiwan University Hospital, 100 Taipei, Taiwan; khchen@ntuh.gov.tw
[4] Division of Pulmonary, Department of Internal Medicine, MacKay Memorial Hospital, 104 Taipei, Taiwan
[5] Department of Medicine, MacKay Medical College, 104 New Taipei City, Taiwan
[6] Department of Internal Medicine, Cardinal Tien Hospital and School of Medicine, College of Medicine, Fu Jen Catholic University, 231 New Taipei City, Taiwan
* Correspondence: Email: stardust.6262@mmh.org.tw (C.-H.C.); email: cywang@mospital.com (C.-Y.W.)

Table S1. Search strategy

Search strategy in Pubmed (Search date: 20210221)

	**6**	**ciprofloxacin**	**30,026**	**03:10:43**
	5	besifloxacin	110	03:10:35
	4	quinolone*	23,479	03:10:28
	3	fluoroquinolone*	24,272	03:10:18
	2	Quinolones [MeSH term]	54,775	03:10:10
**I**	1	Fluoroquinolones [MeSH term]	41,820	03:09:58

Search strategy in Embase (Search date: 20210221)

PICO	No.	Query	Results
**IO**	#33	#21 AND #32	738
	#32	#22 OR #23 OR #31	120427
	#31	#29 AND #30	120427
	#30	#26 OR #27 OR #28	553256
	#29	#24 OR #25	464633
	#28	dilatation	136023
	#27	aneurysm	203042
	#26	dissection	247150
	#25	aortic	389753
	#24	aorta	199797
	#23	'aortic aneurysm'/exp [Emtree term]	64931
**O**	#22	'aortic dissection'/exp [Emtree term]	21119
	#21	#1 OR #2 OR #3 OR #4 OR #5 OR #6 OR #7 OR # 8 OR #9 OR #10 OR #11 OR #12 OR #13 OR #14 OR #15 OR #16 OR #17 OR #18 OR #19 OR #20	215151

	#20	sparfloxacin	4234
	#19	sitafloxacin	982
	#18	pefloxacin	4921
	#17	ofloxacin	27828
	#16	norfloxacin	17626
	#15	nemonoxacin	139
	#14	moxifloxacin	20082
	#13	lomefloxacin	3056
	#12	levofloxacin	39733
	#11	gemifloxacin	1603
	#10	gatifloxacin	6934
	#9	fleroxacin	1877
	#8	enoxacin	3520
	#7	enrofloxacin	6838
	#6	delafloxacin	378
	#5	ciprofloxacin	106739
	#4	besifloxacin	253
	#3	quinolone*	37233
	#2	fluoroquinolone*	24189
**I**	#1	'quinoline derived antiinfective agent'/exp	191336
		**[Emtree term]**	

Search strategy in Cochrane Library (Search date: 20210221)

PICO	Search	Query
**I**	#1	Fluoroquinolones [MeSH term]
	#2	Quinolones [MeSH term]
	#3	fluoroquinolone*
	#4	quinolone*
	#5	besifloxacin
	#6	ciprofloxacin
	#7	delafloxacin
	#8	enrofloxacin
	#9	enoxacin
	#10	fleroxacin
	#11	gatifloxacin
	#12	gemifloxacin
	#13	levofloxacin
	#14	lomefloxacin
	#15	moxifloxacin
	#16	nemonoxacin
	#17	norfloxacin
	#18	ofloxacin
	#19	pefloxacin
	#20	sitafloxacin
	#21	sparfloxacin
	#22	#1 OR #2 OR #3 OR #4 OR #5 OR #6 OR #7 OR #8 OR #9 OR #10 OR #11 OR #12 OR #13 OR #14 OR #15 OR #16 OR #17 OR #18 OR #19 OR #20 OR #21
**O**	#23	Aortic Aneurysm [MeSH term]
	#24	Aneurysm, Dissecting [MeSH term]
	#25	Aorta [MeSH term]
	#26	aortic
	#27	Aneurysm [MeSH term]
	#28	Aneurysm*

	#29	Dissection [MeSH term]
	#30	Dissection*
	#31	dilatation [MeSH term]
	#32	#25 OR #26
	#33	#27 OR #28 OR #29 OR #30 OR #31
	**#34**	**#32 AND #33**
	#35	#23 OR #24 OR #34
**IO**	#36	#22 AND #35

Cochrane Database of Systematic Reviews (CDSR): 5
Cochrane Central Register of Controlled Trials (CENTRAL): 3
Search strategy in Web of Science (Search date: 20210221)

PICO	No.	Query	Results
**IO**	#32	#20 AND #31	93
	#31	#21 OR #22 OR #30	67,704
	#30	#28 AND #29	67,704
	#29	#25 OR #26 OR #27	302,243
	#28	#23 OR #24	282,523
	#27	ALL=(dilatation)	52,348
	#26	ALL=(aneurysm*)	128,778
	#25	ALL=(dissection*)	137,726
	#24	ALL=(aortic)	228,077
	#23	ALL=(aorta)	97,195
	#22	ALL=(aortic aneurysm)	47,478
**O**	#21	ALL=(aortic dissection)	20,156
	#20	#1 OR #2 OR #3 OR #4 OR #5 OR #6 OR #7 OR # 8 OR #9 OR #10 OR #11 OR #12 OR #13 OR #14 OR #15 OR #16 OR #17 OR #18 OR #19	77,559
	#19	ALL=(sparfloxacin)	1,424
	#18	ALL=(sitafloxacin)	304
	#17	ALL=(pefloxacin)	1,333
	#16	ALL=(ofloxacin)	7,905
	#15	ALL=(norfloxacin)	6,338
	#14	ALL=(nemonoxacin)	58
	#13	ALL=(moxifloxacin)	6,431
	#12	ALL=(lomefloxacin)	1,070
	#11	ALL=(levofloxacin)	9,692
	#10	ALL=(gemifloxacin)	645
	#9	ALL=(gatifloxacin)	2,292
	#8	ALL=(fleroxacin)	651
	#7	ALL=(enoxacin)	1,031
	#6	ALL=(enrofloxacin)	4,483
	#5	ALL=(delafloxacin)	137
	#4	ALL=(ciprofloxacin)	34,775
	#3	ALL=(besifloxacin)	129
	#2	ALL=(quinolone*)	20,501
**I**	#1	ALL=(fluoroquinolone*)	23,116

Search strategy in Scopus (Search date: 20210221)

Keywords: ((fluoroquinolone*) OR (quinolone*) OR (besifloxacin) OR (ciprofloxacin) OR (delafloxacin) OR (enrofloxacin) OR (enoxacin) OR (fleroxacin) OR (gatifloxacin) OR(gemifloxacin) OR (levofloxacin) OR (lomefloxacin) OR (moxifloxacin) OR (nemonoxacin) OR (norfloxacin) OR (ofloxacin) OR (pefloxacin) OR (sitafloxacin) OR(sparfloxacin)) AND (((aorta) OR (aortic)) AND ((aneurysm) OR (dissection) OR (dilatation)))

Results: 1764
